# Fast Identification of Adverse Drug Reactions (ADRs) of Digestive and Nervous Systems of Organic Drugs by In Silico Models

**DOI:** 10.3390/molecules26040930

**Published:** 2021-02-10

**Authors:** Meimei Chen, Zhaoyang Yang, Yuxing Gao, Candong Li

**Affiliations:** 1 College of Traditional Chinese Medicine, Fujian University of Traditional Chinese Medicine, Fuzhou 350122, China; chenmeimei1984@163.com (M.C.); yzy813@126.com (Z.Y.); 2 Fujian Key Laboratory of TCM Health Status Identification, Fujian University of Traditional Chinese Medicine, Fuzhou 350122, China; 3 Fujian Engineering Center of Intelligent Diagnosis and Treatment of TCM Four Diagnosis, Fujian University of Traditional Chinese Medicine, Fuzhou 350122, China; 4 College of Chemistry and Chemical Engineering, Xiamen University, Xiamen 361005, China; gaoxingchem@xmu.edu.cn

**Keywords:** adverse drug reactions, drug, QSAR model, SVM, LDA, DL

## Abstract

This study aimed to discover concurrences of adverse drug reactions (ADRs) and derive models of the most frequent items of ADRs based on the SIDER database, which included 1430 marketed drugs and 5868 ADRs. First, common ADRs of organic drugs were manually reclassified according to side effects in the human system and followed by an association rule analysis, which found ADRs of digestive and nervous systems often occurred at the same time with a good association rule. Then, three algorithms, linear discriminant analysis (LDA), support vector machine (SVM) and deep learning, were used to derive models of ADRs of digestive and nervous systems based on 497 organic monomer drugs and to identify key structural features in defining these ADRs. The statistical results indicated that these kinds of QSAR models were good tools for screening ADRs of digestive and nervous systems, which gave the ROC AUC values of 81.5%, 98.9%, 91.5%, 69.5%, 78.4% and 78.8%, respectively. Then, these models were applied to investigate ADRs of 1536 organic compounds with four phase and zero rule-of-five (RO5) violations from the ChEMBL database. Based on the consensus ADRs’ predictions of models, 58.1% and 42.6% of compounds were predicted to cause these two ADRs, respectively, indicating the significance of initial assessment of ADRs in early drug discovery.

## 1. Introduction

Adverse drug reactions (ADRs) are inherent features of drug structures that often caused unnecessary suffering and threats to human health and became obstacles to the discovery of new drugs. In general, drugs have multiple side effects even when prescribed at the appropriate doses and used correctly, as recorded in the SIDER database, which includes information on 1430 marketed drugs and 5868 ADRs [1]. For instance, ibuprofen is the most commonly used and prescribed non-steroidal anti-inflammatory drug. It causes commonly reported side effects, including hemorrhage, vomiting, anemia, decreased hemoglobin, eosinophilia and hypertension [1,2]. Severe ADRs can induce drugs to be withdrawn from the market. For instance, troglitazone, a drug for treatment of diabetes that decreases blood glucose significantly without body weight changes, was withdrawn from the market due to it causing the ADR of hepatic failure [3,4]. Valdecoxib, an anti-inflammatory drug, was withdrawn from the market due to ADRs of nervous and cardiovascular systems [2]. Although ADRs may not be avoided, they can be predictable. Hence, fast identification of ADRs of drugs and filtering out unqualified candidate compounds in early drug discovery play a significant role in the development of new drugs. There are many in vitro methods that screen for toxicological effects, including animal and various cell studies, reactive metabolites, human organ microsomes, etc. [4,5]. However, there are usually some gaps in clinical practice. For instance, the hepatotoxic effects of some drugs in vivo of human and in vitro of animals may be contradictory, such as those of furosemide [6,7]. Differences in acidity and transit time in the gastrointestinal tract between animals and humans can make the solubility and permeability of drugs vary greatly [8]. Additionally, ADRs that impair specific human functions (such as digestive and nervous systems) are difficult to evaluate by these methods. Meanwhile, drugs often have multiple side effects, which may involve some concurrences. Compared with experimental methods, predicting ADRs by in silico models is more time-saving, low-cost and effective.

For the above reasons, human in vivo data are preferable for the identification of associated ADRs of drugs and discovery of their structural characteristics by QSAR modeling, and play a crucial role in filtering out unqualified candidate compounds in early drug discovery. QSAR models apply linear and non-linear algorithms to associate chemical structures with specific activities or properties, which have become increasingly popular in many fields for predicting compound properties, e.g., toxicity, physical properties and biological activity [9]. To date, the QSAR models of hepatotoxicity, cardiotoxicity and nephrotoxicity are commonly reported in light of the relatively clear mechanisms of these ADRs. Pan et al. and Huang et al. developed QSAR models of hepatotoxicity to evaluate hepatotoxicity of traditional Chinese medicines based on the Liver Toxicity Knowledge Base [10,11]. Ancuceanu et al. built computational models for predicting drug hepatotoxicity based on the DILIrank dataset by using machine learning algorithms [12]. Cai et al. built a QSAR model of cardiotoxicity by using a deep learning algorithm for risk assessment of hERG-mediated cardiotoxicities in drug discovery and postmarketing surveillance [13]. Satalkar et al. developed (QSAR) models for fatal drug- induced renal toxicity by using three algorithms, including simple K-means clustering, decision tree and linear regression analysis [14]. Sun et al. developed QSAR classification models to predict potential nephrotoxic ingredients in traditional Chinese medicines by using SVM and ANN [15].

There has not yet been an association analysis of ADRs and QSAR models of ADRs whose mechanisms of action are vague and not easily tested by traditional experimental methods, such as ADRs of digestive and nervous systems. Therefore, this study aimed to discover patterns of concurrences of ADRs among marketed organic drugs, derive models of the most frequent items of ADRs, and mine out key structural features in defining these ADRs based on the SIDER database, which includes 1430 marketed drugs and 5868 ADRs. Firstly, we reclassified common ADRs into seven systematic categories according to side effects in the human system and applied an association rule algorithm named a priori to discover patterns of concurrences of seven ADRs, which found that ADRs of digestive and nervous systems often occurred at the same time with good rule support. Second, three algorithms, linear discriminant analysis (LDA), support vector machine (SVM) and deep learning (DL), were used to derive models of ADRs of digestive and nervous systems based on structures of marketed organic monomer drugs and mine out key structural features in defining these ADRs. Then, these models were simultaneously applied to investigate ADRs of digestive and nervous systems of 1536 organic compounds with four phase and zero rule-of-five (RO5) violations from the ChEMBL database. 

## 2. Results and Discussions

### 2.1. Common ADRs of Marketed Organic Drugs

According to the definition that an ADR is common if occurring at a frequency of greater than 10%, 566 organic drugs were identified as common ADR drugs after removing gold or ion compounds. According to side effects in the human body system, the common ADRs can be grouped into gastrointestinal toxicity, nervous system reaction, allergy, hematopoietic system reaction, circulatory system reaction, hepatotoxicity reaction and urinary system reaction. As listed in Table 1, it was found that 418 drugs had digestive system toxicity, 259 drugs had allergies, and 442 drugs had nervous reactions, 99 drugs caused urinary system reactions, 108 drugs had hematopoietic system reactions, and 75 drugs induced hepatotoxic reactions, respectively.

### 2.2. Associations between ADRs of Organic Drugs

Three key measures of interest for an association rule (support, confidence and lift) were used to select useful rules for the prediction of ADRs. As shown in Table 2, only one association rule was generated, which met the rule of minimum support of 50% and confidence of 80%. The derived association rule was about ADRs of digestive toxicity and nervous reactions, which has the rule support of 59.89%, confidence of 81.1% and lift of 1.04, respectively. This showed that ADRs of digestive and nervous systems often occurred at the same time with the rule support of about 60% and confidence of about 81%. It can be clearly concluded that ADRs of nervous and digestive system were the most frequent item-sets among common ADRs, and ADRs of digestive system can cause ADRs of nervous system, indicating that these two ADRs have some correlations. Thus, it is of great importance to develop QSAR models for understanding and predicting ADRs of nervous and digestive systems, which may eliminate drug candidates with such ADRs in early drug development and reduce the rate of attrition and decrease the cost of drug discovery.

### 2.3. Dataset Splitting

**Figure 1:**
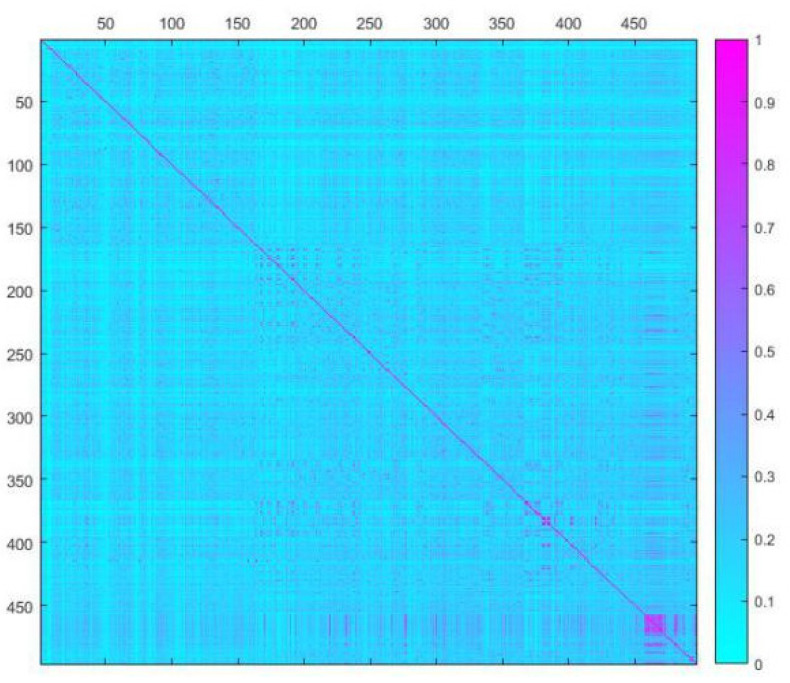
A contour graph of Tanimoto Similarity Index to show the structural similarity of modeling dataset. The abscissa and ordinate are molecular IDs, respectively.

**Figure 2:**
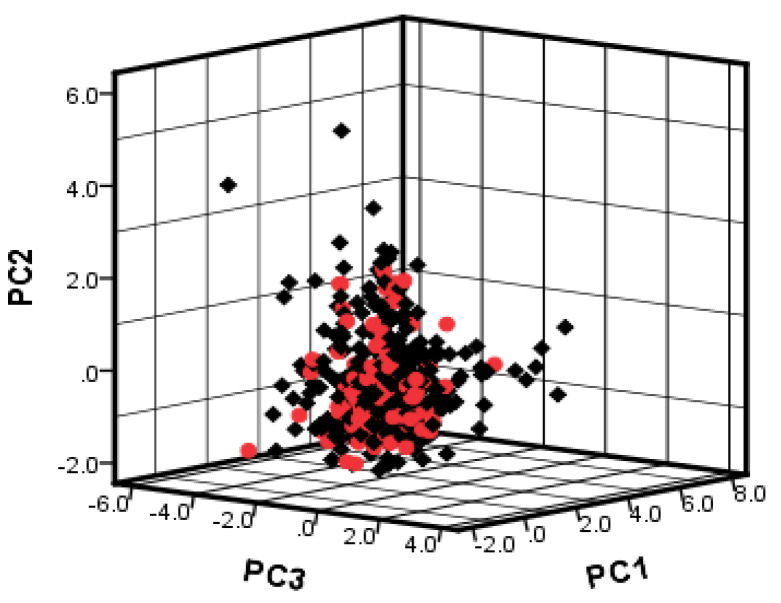
The chemical space distribution of training set and test set based on PC1, PC2 and PC3 components of 130 descriptors. Black diamond represents training set; red round symbol represents test set.

To investigate the chemical diversity of the modeling dataset, the Tanimoto similarity index was calculated based on FP2 fingerprints using Openbabel 2.3.0. Figure 1 demonstrated the Tanimoto similarity indices among drugs ranged from 0.00 to 0.99. From the contour graph of Tanimoto similarity indices, it can be clearly noted that all structures of compounds had low similarity. Additionally, the average index of Tanimoto similarity was only 0.17, which together indicated the significant chemical diversity of modeling dataset. Then, the cluster analysis of 497 drugs was performed by using descriptors and principal component analysis, which led to discovery of 348 clusters of structural diversities. This result further indicated the great structural diversity of the drugs in the dataset. To ensure the biggest structural diversity of the training set, the division of the training and test set chemicals was conducted based on the chemical space distribution of drugs in the cluster analysis. Consequently, 380 drugs were included in the training set and 117 drugs fell into the test set. Figure 2 shows the chemical space distribution of the training set and test set, marked by ‘‘black diamond” and “red round” symbols, respectively.

### 2.4. SW-LDA Model Results of ADRs of Digestive System

After a stepwise method combined with an LDA (SW-LDA) process, 12 molecular descriptors were identified as the QSAR model parameters from the above remaining 130 descriptors. The corresponding LDA model was simultaneously derived by these descriptors. The linear discriminant function was as follows:

y = 0.078 × (PEOE_VSA-4) + 3.495 × PEOE_VSA_FNEG − 3.235 × vsurf_CP + 0.07 × vsurf_DD13 − 1.472 × a_nP + 0.358 × MNDO_LUMO + 0.786 × reactive + 0.768 × a_nI − 0.296 × opr_violation − 2.593 × vsurf_CW1 − 0.007 × SlogP_VSA5 − 0.105 × vsurf_IW7 + 6.105

Table 3 lists the selected descriptors, tolerance, Wilks’ lambda values, variance inflation factor (VIF), F-test values and statistical significance (*p*-value). The statistical significance of all selected descriptors was less than 0.001, showing that they were obvious features in defining ADRs of the digestive system. The tolerance of descriptors was more than 0.1 and VIF was less than 10, indicating no multicollinearity existed among these variables in the LDA model. Table 4 gives the statistical results of the proposed model. As described in Table 4, the obtained LDA model was successful and of good predictive ability. The training accuracy value of 76.58% revealed that the LDA QSAR model can give 76.58% classification accuracy in the training set. The 10-fold cross-validation value of the training set was 72.89% (more than 50%), showing that the developed QSAR LDA model had acceptable stability and predictive ability. Additionally, the predictive accuracy of the test set also reached 72.65%, indicating the good prediction and generalization ability of the LDA model. The sensitivity and specificity of the derived QSAR model were 80.5% and 67.3%, respectively, implying better ability in predicting positive compounds than negative ones. The AUC value of the ROC curve was 81.5%, showing that the LDA model was acceptable in prediction of ADRs of the digestive system and 12 descriptors can be used as the main structural features in defining ADRs of the digestive system.

### 2.5. SW-LDA Model Results of ADRs of Nervous System

After SW-LDA was performed, the best QSAR model for ADRs of the nervous system was generated with six molecular descriptors based on the same training set of ADRs of the digestive system. The obtained QSAR model was given as follows:

y = 0.03 × (PEOE_VSA+5) − 0.244 × vsurf_IW7 − 0.697 × std_dim2 + 0.024 × SMR_VSA3 − 3.498 × Q_VSA_FPPOS + 0.153 × MNDO_dipole + 1.927

The selected variables and their chemical meanings, standard coefficients, tolerance and VIF are shown in Table 5. The values of tolerance, VIF and significance showed that these six descriptors were significant features in defining ADRs of the nervous system and each of them were independent. Table 5 lists the statistical results of the proposed model. As described in Table 4, the derived QSAR model was of acceptable predictive ability. The QSAR model can give 69.21% variance in ADRs of the nervous system in the training set. The accuracy value of 10-fold cross-validation was 68.68% (more than 50%) and the prediction accuracy for the external test set was 64.1% too, showing that the developed QSAR model was of acceptable stability and predictive ability.

### 2.6. Interpretation of the Descriptors 

It is possible to define some vital structural features governing ADRs of digestive or nervous systems by interpreting the molecular descriptors in the QSAR models based on the same training set. In the QSAR models of ADRs of digestive and nervous systems, 12 and 6 descriptors for each model were uncovered, respectively. Additionally, vsurf_IW7 was the same descriptor for two QSAR models, which represents the hydrophilic integy moment that belongs to descriptors of surface area, volume and shape dependent on the structure connectivity and conformation. Here, vsurf_IW7 negatively contributed to these two ADRs, showing that a higher vsurf_IW7 may weaken ADRs of digestive and nervous systems. In order to investigate some correlations between descriptors in two QSAR models, the correlation coefficients of their descriptors were calculated and listed in Table 6. Of note is that, except for the common descriptor (vsurf_IW7), no descriptors in two QSAR models were related to each other based on all correlation coefficient values less than 0.75. This result indicated that vsurf_IW7 was the main factor in defining two ADRs of digestive and nervous systems and other descriptors in two ADR models were independent, especially for descriptors of atom counts and bond counts and physical properties only involved in ADRs of digestive system, including a_nI, a_nP, opr_violation and reactive, and can well distinguish ADRs of the two systems.

### 2.7. Results of SVM Models

The combination of C and γ in RBF kenel function was optimized to derive the best SVM models for ADRs of digestive and nervous systems. The optimum values of C and γ used in the two ADRs models were 150 and 2.5, 150 and 1, together with a maximum 10-fold cross-validation accuracy of 75.79% and 76.05%, respectively. Thereby, the final optimal SVM models for ADRs of digestive and nervous systems were generated as well. As shown in Table 4, the SVM model for ADRs of the digestive system gave quite satisfactory results: accuracy_train_ of 98.42%, accuracy_cv_ of 75.79%, accuracy_test_ of 76.92%, sensitivity of 99.63%, specificity of 95.58% and AUC of 98.9%, respectively, exhibiting significantly high prediction and generalization ability in distinguishing ADRs of digestive system of drugs. Additionally, the final optimal SVM model for ADRs of the nervous system produced satisfactory results: accuracy_train_ of 80.26%, accuracy_cv_ of 76.05%, accuracy_test_ of 83.76% and AUC of 78.4%, respectively. Therefore, compared with the LDA models, SVM models can enhance the ability to predict and generalize the ADRs of digestive and nervous systems.

### 2.8. Results of Deep Learning Models

Similarly, the QSAR models for ADRs of digestive and nervous systems derived by DL were built using the same input variables as used in the above models. Here, we used RapidMiner studio software to perform DL experiments. Table 2 also lists the prediction ability of final DL models of ADRs of digestive and nervous systems. The final DL model of ADRs of digestive system gave satisfactory results: accuracy_train_ of 87.89%, accuracy_cv_ of 78.42%, accuracy_test_ of 78.63%, sensitivity of 94%, specificity of 73.45% and AUC of 91.5%, respectively. Additionally, the final DL model of ADRs of the nervous system gave satisfactory results: accuracy_train_ of 82.89%, accuracy_cv_ of 73.68%, accuracy_test_ of 81.2%, sensitivity of 91.75%, specificity of 53.93% and AUC of 78.8%, respectively. Obviously, DL enhanced the accuracy of descriptors in prediction of ADRs of digestive and nervous systems compared with LDA.

### 2.9. Comparison of Different Approaches and Consensus Prediction

**Figure 3:**
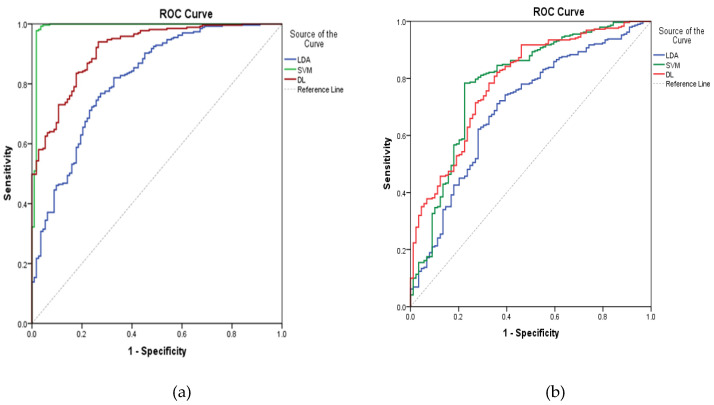
ROC curves for three ADRs of models on digestive (**a**) and nervous systems (**b**).

From the above discussion, three algorithms performed well in prediction of ADRs of digestive and nervous systems based on the same descriptors. Apparently, the performance of SVM in prediction of ADRs of the digestive system outperformed LDA and DL, but in prediction of ADRs of the nervous system, DL was better than SVM from a comprehensive index of ROC curve, as shown in Figure 3, suggesting different algorithms suit peculiar aspects of some special structures. Thus, it seemed reasonable that a consensus predicted result given by these kinds of QSAR models might be more strict and correct than individual models. Here, a consensus prediction of ADRs of digestive and nervous systems was derived by averaging the predictions for the dataset given by the individual models [15]. Based on the consensus ADR predictions of 1536 organic compounds with four phase and zero RO5 violations from ChEMBL database by three models, we found that 893 and 654 compounds were computationally identified to cause the above two ADRs, respectively. Among them, 433 compounds were predicted to cause these two ADRs. These results can be seen in the supplementary materials, indicating the significance of initial assessment of ADRs in early drug discovery.

## 3. Methods and Materials

### 3.1. Association Analysis of Common Side Effects of Drugs

As defined in the SIDER database, ADRs with a frequency of more than 10% were identified as common ADRs and then grouped in seven systematic categories according to side effects in the human body system, including digestive, nervous, hepatotoxic, urinary, allergy, circulatory and hemopoietic systems. Additionally, we removed non-organic compounds that could not be further analyzed by descriptor calculation, which led to an acquisition of 566 organic drugs with common ADRs. Then, to explore the correlations between multiple side effects of organic drugs, an association rule analysis was applied to discover patterns of concurrences of ADRs in the database. Support, confidence and lift are three key measures of interestingness of an association rule. Support is an indicator of rule frequency. Confidence is the probability that consequent B will follow antecedent A. Lift is an indicator of the contribution antecedent A makes to consequent B [16]. Here, the association algorithm named a priori embedded in the clementine 12 software (SPSS, Inc., Chicago, IL, USA) was performed to search for concurrences of ADRs. The maximum frequent set was fixed to no more than 5, the minimum support and confidence of rules were set to 50% and 80%, respectively.

### 3.2. Molecular Descriptor Calculation

First, the molecular structures of 566 organic marketed drugs with common ADRs were downloaded from the Pubchem database based on their names and checked one by one. After removing multi-compound drugs, a total of 497 organic monomer drugs remained. Then, they were put into the Molecular Operating Environment software (MOE2008.10, Chemical Computing Group Inc., Montreal, Canada), to be subjected to the energy minimization of 3D structures. Subsequently, stochastic conformation search was performed to optimize their conformer structures. Then, a total of 327 diverse descriptors of optimized structures were calculated by utilizing the QSAR module of MOE. These 327 descriptors consisted of 184 2D molecular properties, 86 i3D molecular properties and 10 x3D structural information, which may be redundant and irrelevant for QSAR development. Thus, the constant or almost constant descriptors for all molecules were first deleted and then a pairwise correlation analysis was conducted to remove one of inter-correlated descriptors (with a correlation coefficient value greater than 0.95) [17]. Finally, a total set of 130 descriptors remained and was used for QSAR modeling.

### 3.3. Data Splitting

To investigate the chemical diversity of the whole dataset, the Tanimoto similarity index was calculated based on FP2 fingerprints using Openbabel 2.3.0 [18]. The Tanimoto similarity coefficient is the atomic pair shared between two molecules divided by all their atomic pairs, defined as c/(a + b + c). The variable c is the number of atomic pairs of the two compounds, and a and b are the numbers of their unique atomic pairs. To obtain reliable QSAR models, the data set was split into a training set and a test set by a range ratio of 3:1~4:1. The training set was used to construct QSAR models, and the test set was used as an external validation of derived models. To ensure the training set spanned the whole descriptor space and kept a balance distribution of the chemicals in two data sets [19], the cluster analysis of dataset was further investigated using the QuaSAR-Cluster module in MOE, which calculated the descriptor average vector x0 and covariance matrix S based on principal component analysis to assign similar molecules to one cluster. 

### 3.4. QSAR Model Approach

#### 3.4.1. Stepwise Linear Discriminant Analysis

LDA is one of the data dimensionality reduction and classification techniques widely used in QSAR modeling. The basic idea of the LDA algorithm is to project the data in low dimensions, so that the projection centers of the same type of data are as close as possible, and the projection centers of different types of data are as far apart as possible [20]. Feature selection is one important step for development of QSAR models. In this study, a stepwise method combined with LDA (SW-LDA) was conducted to derive the QSAR models and mine significant features in defining ADRs of drugs, which used F-test to eliminate redundant variables at each stage of the descriptor selection. Here, we used the default set of F values (Fmax = 3.84 and Fmin = 2.71) in the SW-LDA algorithm embedded in Clementine 12. 

#### 3.4.2. Support Vector Machine (SVM)

SVM is a very classic and efficient classification and regression algorithm proposed by Vapnik et al. in 1998 [21]. Compared with the current mainstream deep neural network technology, the SVM algorithm has certain advantages in solving small sample, nonlinear and high-dimensional feature data pattern recognition problems. The core idea of the SVM algorithm is to find the optimal classification surface (also called the hyperplane) between the two classes. SVM uses kernel functions such as the radial basis function (RBF), spline and Bessel for nonlinear transformation of the input space. Here, the RBF kernel in the SVM algorithm embedded in Clementine 12 was performed to derived non-linear models [22].

#### 3.4.3. Deep Learning (DL)

Deep learning is based on a multi-layer feed-forward artificial neural network that is trained with stochastic gradient descent using back-propagation [23]. The network can contain a large number of hidden layers consisting of nervous with tanh, rectifier or maxout activation functions [24]. Advanced features such as adaptive learning rate, rate annealing, momentum training, dropout and L1 or L2 regularization enable high predictive accuracy. Each compute node trains a copy of the global model parameters on its local data with multi-threading (asynchronously), and contributes periodically to the global model via model averaging across the network. Here, the DL algorithm embedded in the RapidMiner studio software (education version, RapidMiner, Inc., Boston, MA, USA) was conducted to derive QSAR models by using the maxout activation function.

### 3.5. Performance Evaluation

Then, to evaluate the predictive ability and reliability of QSAR models, widely applied internal and external validations, such as the 10-fold cross-validation and the test set validation, were applied. In the 10-fold cross-validation, the training set is randomly divided into ten equal subsets. Each time, one of the ten subsets is used as the validation set and the other nine subsets are put together to build a model. Then the average error across all ten trials is computed. Further, four important evaluation indicators for performance of QSAR models, including accuracy (ACC), balanced accuracy (BACC), sensitivity (SE), and specificity (SP), were calculated as follows [25].

$$\begin{array}{c} \mathrm{ACC}=\left(\mathrm{TP}+\mathrm{TN}\right)/\left(\mathrm{TP}+\mathrm{TN}+\mathrm{FP}+\mathrm{FN}\right),\\ \mathrm{SE}=\mathrm{TP}/\left(\mathrm{TP}+\mathrm{FN}\right),\\ \mathrm{SP}=\mathrm{TN}/\left(\mathrm{TN}+\mathrm{FP}\right),\\ \mathrm{BACC}=\left(\mathrm{SE}+\mathrm{SP}\right)/2, \end{array}$$

where TP, TN, FP and FN represent the number of true positive, true negative, false positive and false negative ones, respectively. Additionally, the receiver operating characteristic (ROC) curve was performed to evaluate QSAR models with a more global and unbiased evaluation, which is a comprehensive index reflecting sensitivity and specificity [26]. The ROC curve is a graphical plot of the sensitivity or true positive rate against the false positive rate (1-specificity), which can be quantitatively described by the area under the curve (AUC). The larger the AUC, the higher the diagnostic accuracy.

### 3.6. Model Application

To investigate ADRs of digestive and nervous systems of other drugs, we applied these models in prediction of 1536 organic compounds with four phase and zero RO5 violations in the ChEMBL database [27], respectively. To obtain more strict and correct results, a consensus prediction of ADRs of digestive and nervous systems was derived by all three models, respectively.

## 4. Conclusions

In this study, the association rule analysis was initially used to discover patterns of concurrences of ADRs of 566 marketed organic drugs in the SIDER database. Then, three QSAR modeling algorithms, LDA, SVM and DL, were successfully used to derive models of ADRs of digestive and nervous systems and identified key structural features in defining these ADRs of organic drugs based on 497 marketed organic monomer drugs. Satisfactory results were obtained as follows. First, ADRs of digestive and nervous systems often occurred at the same time with the rule support of about 60% and confidence of about 81%, indicating these two ADRs co-occurred very frequently in adverse drug events. Second, QSAR models derived by LDA, SVM and DL were good tools for screening ADRs of digestive and nervous systems, which gave the ROC AUC values of 81.5%, 98.9%, 91.5%, 69.5%, 78.4% and 78.8% in discriminating ADRs of digestive and nervous systems, respectively. The vsurf_IW7 was the same descriptor for two QSAR models, which may be responsible for two ADRs of digestive and nervous systems. Other descriptors in two ADR models were independent, especially for descriptors of atom counts and bond counts and physical properties only involved in ADRs of the digestive system, including a_nI, a_nP, opr_violation and reactive, and can well distinguish these two ADRs. Then, these models were applied to investigate ADRs of digestive and nervous systems of 1536 organic compounds with four phase and zero RO5 violations from the ChEMBL database. Based on the consensus ADR predictions of models, among 1536 organic compounds 58.1% and 42.6% of compounds were computationally identified to cause such two ADRs, respectively, indicating the significance of initial assessment of ADRs in early drug discovery.

## Figures and Tables

**Table 1:** Some common adverse drug reaction (ADR) information on drugs in the SIDER database.

ID	ADR Categories	Main Items of ADRs	No. of Drugs
1	Digestive system reactions	Abdominal pain; diarrhea; abdominal bloating; constipation; gastrointestinal disorder; nausea; anorexia; digestion impaired; gastrointestinal hemorrhage; flatulence; abnormal feces; abdominal pain upper; vomiting; gastroesophageal reflux disease, etc.	418
2	Nervous reactions	Abnormal involuntary movements; convulsion; headache; balance disorder; dizziness; arthralgia; depression; paresthesia; ataxia; amnesia; disorder sight; feeling abnormal; deafness; abnormal behavior; nervous symptoms; etc.	442
3	Allergy reactions	Pruritus; dermatitis; dyspnea; injection site pain; rash maculo-papular; erythema; acne; application site irritation; anaphylactic shock; injection site reaction; application site erythema; allergic contact dermatitis; seborrheic dermatitis; blisters; etc.	259
4	Hepatotoxic reactions	Alanine aminotransferase increased; liver function test abnormal; hepatic encephalopathy; hepatic enzyme abnormal; transaminases increased; hepatitis; hepatotoxicity; hepatic failure; etc.	75
5	Cardiovascular reactions	Atrial fibrillation; cardiac output decreased; bradycardia; angina pectoris; arrhythmia; acute coronary syndrome; arterial insufficiency; cardiac disorder; angiopathy; atrial fibrillation; cardiac murmur; cardiotoxicity; blood triglycerides increased; ventricular arrhythmia; etc.	85
6	Urinary reactions	Urinary tract infection; dysuria; bladder pain; micturition disorder; urinary hesitation; nephropathy toxic; renal failure; renal tubular acidosis; blood creatinine increased; urinary retention; albuminuria; hematuria; urethral disorder; chronic kidney disease; protein in urine; etc.	99
7	Hematologic reactions	Thrombocytopenia; anemia; coagulopathy; agranulocytosis; eosinophilia; hemoglobin decreased; platelet count decreased; activated partial thromboplastin time prolonged; white blood cell count decreased; etc.	108

**Table 2:** Results of association rule analysis of ADRs of organic drugs.

Consequent	Antecedent	Instances	Support%	Confidence%	Rule Support%	Lift
ADRs of nervous system	ADRs of digestive system	418	73.85	81.1	59.89	1.04

**Table 3:** Molecular descriptors and the standardized coefficient of the linear discriminant analysis (LDA) model for ADRs of digestive system.

Descriptors	Chemical Meaning	Tolerance	Wilks’ Lambda	VIF	F to Remove	Non-Standardized Coefficient	*p*-Value
PEOE_VSA-4	Total negative van der Waals surface area	0.771	0.853	1.297	71.489	0.078	0.00
PEOE_VSA_FNEG	Fractional negative van der Waals surface area	0.773	0.736	1.294	11.259	3.495	0.00
vsurf_CP	Critical packing parameter	0.697	0.745	1.435	15.798	−3.235	0.00
vsurf_DD13	Contact distances of lowest hydrophobic energy	0.908	0.737	1.101	11.578	0.07	0.00
a_nP	Number of phosphorus atoms	0.898	0.74	1.114	13.367	−1.472	0.00
MNDO_LUMO	The energy (eV) of the lowest unoccupied molecular orbital calculated using the MNDO Hamiltonian	0.757	0.75	1.321	18.153	0.358	0.00
reactive	Indicator of the presence of reactive groups	0.95	0.733	1.052	9.782	0.786	0.00
a_nI	Number of iodine atoms	0.972	0.728	1.029	7.317	0.768	0.00
opr_violation	The number of violations of Oprea’s lead-like test	0.418	0.739	2.392	12.911	−0.296	0.00
vsurf_CW1	Capacity factor	0.417	0.74	2.392	13.506	−2.593	0.00
SlogP_VSA5	The subdivided surface areas	0.715	0.725	1.399	5.435	−0.007	0.00
vsurf_IW7	Hydrophilic integy moment	0.946	0.723	1.057	4.663	−0.105	0.00
Constant						6.105	

**Table 4:** The classification performance of the derived QSAR models for ADRs of digestive and nervous systems.

QSAR Models		Accuracy Training Set	Accuracy Test Set	Accuracy 10-Fold CV	Total Accuracy	BACC	Sensitivity	Specificity	ROC AUC
Digestive system	LDA	76.58%	72.65%	72.89%	75.65%	73.9%	80.5%	67.3%	0.815
	SVM	98.42%	76.92%	75.79%	93.36%	97.61%	99.63%	95.58%	0.989
	DL	87.89%	78.63%	78.42%	85.71%	83.73%	94%	73.45%	0.915
Nervous system	LDA	69.21%	64.1%	68.68%	68%	67.4%	70.8%	64%	0.695
	SVM	80.26%	83.76%	76.05%	81.09%	62.94%	95.53%	30.34%	0.784
	DL	82.89%	81.2%	73.68%	82.49%	72.84%	91.75%	53.93%	0.788

**Table 5:** Molecular descriptors and the standardized coefficient of the LDA model for ADRs of nervous system.

Descriptors	Chemical Meaning	Tolerance	Wilks’ Lambda	VIF	F to Remove	Non-standardized Coefficient	*p*-Value
PEOE_VSA+5	Total positive van der Waals surface area	0.53	0.932	1.887	7.712	0.03	0.004
vsurf_IW7	Hydrophilic integy moment	0.949	0.932	1.054	7.875	−0.244	0.001
std_dim2	Standard dimension 2	0.311	0.939	3.215	10.599	−0.697	0.00
SMR_VSA3	Subdivided surface areas	0.304	0.933	3.289	8.231	0.024	0.00
Q_VSA_FPPOS	Fractional positive polar van der Waals surface area	0.917	0.925	1.091	4.821	−3.498	0.00
MNDO_dipole	The dipole moment calculated using the MNDO Hamiltonian	0.888	0.923	1.126	4.261	0.153	0.00
Constant						1.927	

**Table 6:** Correlation coefficients of descriptors in two QSAR models of ADRs of digestive and nervous systems.

Descriptors	PEOE_ VSA+5	Q_VSA_ FPPOS	MNDO_ dipole	SlogP_ VSA3	vsurf_ IW7	std_ dim2
reactive	0.127	−0.027	0.058	0.169	−0.078	0.129
a_nI	0.108	0.056	−0.073	0.075	−0.054	0.049
a_nP	−0.044	0.166	-0.022	0.036	−0.021	0.038
PEOE_VSA-4	−0.047	0.173	0.143	0.014	0.002	−0.018
PEOE_VSA_FNEG	−0.071	−0.714	−0.076	−0.244	0.131	−0.131
opr_violation	0.453	0.152	−0.205	0.488	−0.109	0.698
MNDO_LUMO	−0.001	0.05	−0.134	0.014	−0.007	0.004
SlogP_VSA5	0.253	0.268	−0.131	0.292	−0.012	0.412
vsurf_CP	−0.263	−0.4	−0.107	−0.203	0.086	−0.211
vsurf_CW1	−0.308	0.247	0.23	−0.373	−0.038	−0.591
vsurf_DD13	0.415	0.053	−0.163	0.499	−0.008	0.47
vsurf_IW7	−0.07	−0.185	0.102	−0.08	1	−0.074

## Data Availability

The data presented in this study are available within the article and in supplementary materials.

## Supplementary Materials

The following are available online: ADRs predictions of 1536 organic compounds with four phase and zero rule-of-five (RO5) violations from the ChEMBL database.

## References

[1] Michael K., Ivica L., Juhl J.L., Peer B. (2016). The SIDER database of drugs and side effects. Nucleic Acids Res..

[2] DrugBank. https://www.drugbank.ca/..

[3] Watkins P.B. (2005). Insight into hepatotoxicity: The troglitazone experience. Hepatology.

[4] Kuna L., Bozic I., Kizivat T., Bojanic K., Mrso M., Kralj E., Smolic R., Wu G.Y., Smolic M. (2018). Models of drug induced liver injury (DILI)—Current issues and future perspectives. Curr. Drug Metab..

[5] Hosey C.M., Benet L.Z., Chackalamannil S., Rotella D., Ward S. (2017). Experimental ADME and Toxicology. Comprehensive Medicinal Chemistry III.

[6] FDA (2009). Guidance for industry integrated summaries of effectiveness and safety: Location within the common technical document.

[7] Walker R.M., McElligott T.F. (1981). Furosemide induced hepatotoxicity. J. Pathol..

[8] Markovic M., Zur M., Fine-Shamir N., Haimov E., González-Álvarez I., Dahan A. (2020). Segmental-dependent solubility and permeability as key factors guiding controlled release drug product development. Pharmaceutics.

[9] Lo Piparo E., Fratev F., Lemke F., Mazzatorta P., Smiesko M., Fritz J.I., Benfenati E. (2016). QSAR models for Daphnia magna toxicity prediction of benzoxazinone allelochemicals and their transformation products. J. Agric. Food Chem..

[10] Zhao P., Liu B., Wang C. (2017). Hepatotoxicity evaluation of traditional Chinese medicines using a computational molecular model. Clin. Toxicol..

[11] Huang S.H., Tung C.W., Fülöp F., Li J.H. (2015). Developing a qsar model for hepatotoxicity screening of the active compounds in traditional Chinese medicines. Food Chem Toxicol..

[12] Ancuceanu R., Hovanet M.V., Anghel A.I., Furtunescu F., Dinu M. (2020). Computational models using multiple machine learning algorithms for predicting drug hepatotoxicity with the dilirank dataset. Int. J. Mol. Sci..

[13] Cai C., Guo P., Zhou Y., Zhou J., Wang Q., Zhang F., Fang J., Cheng F. (2019). Deep learning-based prediction of drug-induced cardiotoxicity. J. Chem. Inf. Model.

[14] Satalkar V., Kulkarni S., Joshi D. (2015). QSAR based analysis of fatal drug induced renal toxicity. J. Comput. Methods Mol. Des..

[15] Sun Y., Shi S., Li Y., Wang Q. (2019). Development of quantitative structure-activity relationship models to predict potential nephrotoxic ingredients in traditional chinese medicines. Food Chem. Toxicol..

[16] Ordonez C., Zhao K. (2011). Evaluating association rules and decision trees to predict multiple target attributes. Intell. Data Anal..

[17] Chen M., Yang X., Lai X., Gao Y. (2016). Structural Investigation for optimization of anthranilic acid derivatives as partial fxr agonists by in silico approaches. Int. J. Mol. Sci..

[18] O’Boyle N.M., Banck M., James C.A., Morley C., Vandermeersch T., Hutchison G.R. (2011). Open Babel: An open chemical toolbox. J. Cheminform..

[19] Chen M., Yang F., Kang J., Gan H., Lai X., Gao Y. (2018). Discovery of molecular mechanism of a clinical herbal formula upregulating serum HDL-c levels in treatment of metabolic syndrome by in vivo and computational studies. Bioorg. Med. Chem. Lett..

[20] Guo J. (2010). Simultaneous variable selection and class fusion for high-dimensional linear discriminant analysis. Biostatistics.

[21] Vapnik V. (1998). The Support Vector Method of Function Estimation. Nonlinear Modeling.

[22] Cheng F., Guo T., Liu C., Wang Y., Huang B. (2019). Identification of the thief zone using a support vector machine method. Processes.

[23] LeCun Y., Bengio Y., Hinton G. (2015). Deep learning. Nature.

[24] Khumprom P., Yodo N. (2019). A data-driven predictive prognostic model for lithium-ion batteries based on a deep learning algorithm. Energies.

[25] Zhang Y., Yang S., Liu Y., Zhang Y., Zhou F. (2018). Integration of 24 feature types to accurately detect and predict seizures using scalp EEG signals. Sensors.

[26] Linden A. (2006). Measuring diagnostic and predictive accuracy in disease management: An introduction to receiver operating characteristic (ROC) analysis. J. Eval. Clin. Pract..

[27] Bento A.P., Gaulton A., Hersey A., Bellis L.J., Chambers J., Davies M., Krüger F.A., Light Y., Mak L., McGlinchey S. (2014). The ChEMBL bioactivity database: An update. Nucleic Acids Res..

